# Interaction between coronaviruses and the autophagic response

**DOI:** 10.3389/fcimb.2024.1457617

**Published:** 2024-11-22

**Authors:** Jiarong Yu, Shengqiang Ge, Jinming Li, Yongqiang Zhang, Jiao Xu, Yingli Wang, Shan Liu, Xiaojing Yu, Zhiliang Wang

**Affiliations:** ^1^ China Animal Health and Epidemiology Center, Qingdao, China; ^2^ College of Veterinary Medicine, Qingdao Agricultural University, Qingdao, China

**Keywords:** coronavirus, SARS-CoV-2, autophagy, virus replication, immune response

## Abstract

In recent years, the emergence and widespread dissemination of the coronavirus SARS-CoV-2 has posed a significant threat to global public health and social development. In order to safely and effectively prevent and control the spread of coronavirus diseases, a profound understanding of virus-host interactions is paramount. Cellular autophagy, a process that safeguards cells by maintaining cellular homeostasis under diverse stress conditions. Xenophagy, specifically, can selectively degrade intracellular pathogens, such as bacteria, fungi, viruses, and parasites, thus establishing a robust defense mechanism against such intruders. Coronaviruses have the ability to induce autophagy, and they manipulate this pathway to ensure their efficient replication. While progress has been made in elucidating the intricate relationship between coronaviruses and autophagy, a comprehensive summary of how autophagy either benefits or hinders viral replication remains elusive. In this review, we delve into the mechanisms that govern how different coronaviruses regulate autophagy. We also provide an in-depth analysis of virus-host interactions, particularly focusing on the latest data pertaining to SARS-CoV-2. Our aim is to lay a theoretical foundation for the development of novel coronavirus vaccines and the screening of potential drug targets.

## 1 Introduction

In 2019, Wuhan, Hubei, China, witnessed the emergence of a novel coronavirus, dubbed COVID-19, whose exceptionally contagious nature triggered a rapid dissemination of the disease across the globe, persisting as a pandemic in the epicenter of the outbreak (Huang et al., 2020; Zhu et al., 2020). Subsequently, the Coronavirus Study Group of the International Committee on Taxonomy of Viruses (ICTV) categorized this virus under the β-coronavirus genus, designating it as Severe Acute Respiratory Syndrome Coronavirus 2 (SARS-CoV-2). As of December 2021, global statistics indicate a staggering 271 million confirmed cases and 5.32 million fatalities, while a viable vaccine against SARS-CoV-2 remains elusive. Furthermore, coronaviruses pose a significant threat to economic animals, inflicting immense economic losses on the global livestock industry.

### 1.1 Introduction to coronaviruses

The coronavirus is classified under the Nidovirales order and falls within the Orthocoronavirus subfamily of the Coronavirus family (CoV). This expansive family is further segmented into four distinct viral genera: Alphacoronavirus (Alpha-CoV, α-CoV), Betacoronavirus (Beta-CoV, β-CoV), Gammacoronavirus (Gamma-CoV, γ-CoV), and Deltacoronavirus (Delta-CoV, δ-CoV) (Schoeman and Fielding, 2019). Coronaviruses possess a diverse range of hosts, encompassing mammals, birds, rodents, ruminants, and more. These infections in hosts can trigger a spectrum of illnesses, including respiratory disease, intestinal disease, hepatitis, and neurological disorders. Notably, α-CoV and β-CoV predominantly target mammals, while γ-CoV and δ-CoV predominantly affect avian species (Gorbalenya et al., 2004; Woo et al., 2010; Woo et al., 2012). Here, we offer a concise overview of the specific characteristics associated with the various coronaviruses (**Table 1**).

**Table 1:** Classification, host and clinical symptoms of coronaviruses.

Subgenus	Modern name	Host	Clinical symptom	Reference
α-CoV	Human coronavirus 229E (HCoV-229E)	Human	The predominant symptoms of respiratory illness include general malaise, headache, a runny nose, frequent sneezing, a sore throat, and fever.	(Hamre and Procknow, 1966; Hendley et al., 1972; Wenzel et al., 1974)
	Human coronavirus NL63 (HCoV-NL63)	Human	Minor symptoms of respiratory illness, such as coughing, asthma-like symptoms, fever, and hypoxia, tend to resolve spontaneously over time.	(Arden et al., 2005; Chiu et al., 2005; van der Hoek et al., 2005; Abdul-Rasool and Fielding, 2010)
	Porcine epidemic diarrhea virus (PEDV)	Pig	This virus has the ability to infect pigs of various ages, causing severe diarrhea, vomiting, and dehydration. Notably, lactating piglets younger than two weeks of age experience particularly severe clinical symptoms, resulting in a mortality rate of 100% among these piglets.	(Wood, 1977; Coussement et al., 1982)
	Transmissible gastroenteritis virus (TGEV)	Pig	The infection is often accompanied by a loss of appetite and diarrhea, leading to a staggering 100% mortality rate among piglets under two weeks of age.	(Doyle and Hutchings, 1946; Xia et al., 2018)
	Swine acute diarrhea syndrome-coronavirus (SASD-CoV)	Pig	The clinical symptoms exhibited are analogous to those of PEDV, albeit with vomiting, diarrhea, and dehydration manifesting at a later stage.	(Gong et al., 2017; Pan et al., 2017)
	Porcine respiratory coronavirus (PRCV)	Pig	Only mild or subclinical symptoms are observed, with no occurrence of severe respiratory symptoms or fatalities.	(Van Nieuwstadt et al., 1989)
	Feline coronavirus (FCoV)	Cat	The most prominent symptoms of the exudative type are due to fibrinous or granulomatous plasmacytitis, leading to abdominal enlargement with a watery fluctuating sensation, fever, depression, diarrhea, respiratory distress, and anemia. In contrast, the most characteristic signs of the non-exudative type are corneal edema and confusion, hemorrhage and pus accumulation in the eye chamber, vision loss, and in some instances, neurological manifestations such as ataxia and mild paralysis.	(Andrew, 2000; Doki et al., 2018)
	Canine coronavirus (CCoV)	Dog	Affected dogs exhibit symptoms such as lethargy, loss of appetite, vomiting, diarrhea, severe dehydration, and so forth. Puppies are particularly vulnerable, with a high mortality rate, and may experience recurrences post-treatment. However, adult dogs exhibit a stronger resistance, often manifesting only mild diarrhea that typically resolves by itself.	(Tennant et al., 1991; Decaro and Buonavoglia, 2008)
β-CoV	Severe acute respiratory syndrome coronavirus (SARS-CoV)	Human	Infected patients initially exhibit symptoms such as fever, headache, and muscle pain, which then progress to include cough, dyspnea, and respiratory distress. Furthermore, some patients develop diarrhea, and imaging studies reveal diffuse alveolar damage to the lungs of those infected with SARS-CoV.	(Lee et al., 2003; Peiris et al., 2003; Wong et al., 2003; Tee et al., 2004)
	Middle East respiratory syndrome-related coronavirus (MERS-CoV)	Human	Initially, the patient exhibits symptoms of fever, cough, sore throat, muscle pain, and joint pain. Subsequently, these symptoms are followed by respiratory distress, pneumonia, multi-organ failure affecting the kidneys and heart, ultimately leading to death. Additionally, some patients develop gastrointestinal disorders such as vomiting and diarrhea, alongside symptoms of renal insufficiency and viraemia.	(Van Boheemen et al., 2012; Arabi et al., 2014; Lin et al., 2014; Zumla et al., 2015)
	Severe acute respiratory syndrome coronavirus 2 (SARS-CoV-2)	Human	Infected patients exhibit a wide spectrum of symptoms, ranging from mild manifestations to severe respiratory failure. Initially, patients develop cough, fever, myalgia, diarrhea, and lymphopenia. In severe cases, however, viral sepsis and lung damage emerge, leading to complications such as pneumonia, respiratory failure, coagulation disorders, shock, renal and hepatic damage, central nervous system impairment, multiple organ damage, and ultimately, death.	(Chan et al., 2020; Chen et al., 2020; Huang et al., 2020; Li et al., 2020; Wu and McGoogan, 2020; Zheng, 2020)
	Human coronavirus OC43 (HCoV-OC43)	Human	Patients initially present with symptoms such as cough, fever, sore throat, headache, lung rales, pneumonia, and fine bronchitis.	(Vabret et al., 2003; Dominguez et al., 2009; Liu et al., 2021)
	Human coronavirus HKU1 (HCoV-HKU1)	Human	Infected individuals exhibit symptoms including nasal discharge, cough, fever, sore throat, chills, and enlarged tonsils. In addition, some patients may progress to develop pneumonia and fine bronchitis.	(Woo et al., 2005a; Woo et al., 2005b; Woo et al., 2009)
	Bovine coronavirus (BCoV)	Cattle	Young cows displayed symptoms such as cupping, diarrhea, dehydration, acidemia, and ultimately death. Among infected adult cows, there were signs of profuse diarrhea, weight loss, depression, and decreased milk production. Additionally, some cows exhibited coughing, a runny nose, and an increased respiratory rate.	(Woode et al., 1978; Kruiningen et al., 1985; Durham et al., 1989)
	Equine coronavirus (ECoV)	Horse	Infected horses exhibit anorexia, depression, fever, diarrhea, and neurological manifestations, including ataxia and recumbency.	(Pusterla et al., 2013; Fielding et al., 2015; Giannitti et al., 2015)
	Porcine hemagglutinating encephalomyelitis virus (PHEV)	Pig	The virus can infect pigs of all ages, with adult pigs exhibiting subclinical symptoms, while some sows may temporarily lose their appetite. On the other hand, piglets infected display muscle tremors, encephalomyelitis, coughing, vomiting or dry heaves, diarrhea, dehydration, respiratory distress, coma, and ultimately, death.	(Lorbach et al., 2017; Greig and Girard, 1969; Rho et al., 2011; Mora-Díaz et al., 2019)
	Murine hepatitis virus (MHV)	Mouse	Infected rats exhibit signs of pneumonia characterized by interstitial lung infiltrates, congestion, and haemorrhage. Additionally, abdominal or intravenous infections in certain rats can induce liver disease, while oral ingestion results in gastrointestinal symptoms and hepatitis.	(Lavi et al., 1986; Haring and Perlman, 2001; De Albuquerque et al., 2006)
γ-CoV	Infectious bronchitis virus (IBV)	Chicken	Sick chickens may exhibit symptoms such as depression, respiratory distress, wheezing, coughing, lethargy, watery eyes, and mild sinus swelling. Roosters infected may manifest signs of infertility, while hens display a decrease in both the quality and quantity of eggs produced. Furthermore, infected laying hens tend to have reduced egg production.	(Cook and Mockett, 1995; Fadly, 2008; Ganapathy, 2009)
	Turkey coronavirus (TCoV)	Turkey	Infected turkeys commonly suffer from depression, diarrhea, dehydration, and stunted growth. Similarly, adult chickens exhibit a notable decrease in meat or egg production when infected.	(Guy, 2000; Ismail et al., 2003)
δ-CoV	Porcine deltacoronavirus (PDCoV)	Pig	Sick pigs manifest severe symptoms including diarrhea, vomiting, dehydration, and ultimately, death.	(Li et al., 2014; Song et al., 2015; Janetanakit et al., 2016)
	Human Porcine deltacoronavirus (Hu-PDCoV)	Human	Patients typically present with symptoms such as fever, cough, abdominal pain, and diarrhea.	(Lednicky et al., 2021)

Coronavirus is a single-stranded, positive-sense RNA virus that is enveloped and possesses a genome size ranging approximately from 24,500 to 31,800 bp (Mihindukulasuriya et al., 2008). Its genome is characterized by a cap structure at the 5’ end and a polyadenylated tail at the 3’ end, sandwiched between which lies an intermediate structure composed of seven overlapping open reading frames (ORFs). These ORFs encode three primary non-structural proteins: replicase proteins (ORF1a and ORF1b), the auxiliary protein ORF3, and four structural proteins: spike protein (S), envelope protein (E), membrane protein (M), and nucleocapsid protein (N). The genes encoding ORF1a and ORF1b constitute approximately two-thirds of the entire genome and can be further dissected into 15-16 non-structural proteins (nsp), primarily involved in viral replication processes (Wang et al., 2020). The initial strategy employed by the coronavirus to circumvent the host’s immune defense system involves suppressing the production of IFNα/β, marking a crucial juncture for the virus to achieve successful replication. Nsp1 facilitates mRNA degradation and impedes host cell protein translation, thereby suppressing the innate immune response (Nakagawa and Makino, 2021). Among these nsps, Nsp3, the largest multi-domain protein, harbors a PLPro/Deubiquitinase structural domain that cleaves viral polyproteins and mitigates the innate immune response (Imbert et al., 2008; Neuman et al., 2014). The coronavirus establishes a “replication factory” known as a double-membrane vesicle (DMV), where Nsp3 and Nsp4 play pivotal roles in its formation. This DMV serves as a secure environment for viral replication, shielding viral RNA and encapsulating replication complexes to evade detection by host sensors (Knoops et al., 2008; Oudshoorn et al., 2017). Furthermore, Nsp15 facilitates viral genome replication and transcription by reducing the accumulation of negative-strand RNA and dsRNA, thereby aiding in the evasion of detection by host RNA sensors (Gao et al., 2021). Nsp16 possesses 2′-O-methyltransferase activity, enabling viral mRNA to mimic host mRNA and thus escape the interferon (IFN) response (Züst et al., 2011). Additionally, the coronavirus targets the double-stranded RNA sensor (RIG-I) and melanoma differentiation-associated protein 5 (MDA5). Notably, the N protein of SARS-CoV, SARS-CoV-2, and MERS-CoV interacts with TRIM25 E3 ubiquitin ligase, disrupting the K63-linked ubiquitination of RIG-I and preventing its activation (Chang et al., 2020; Chen et al., 2020; Xue et al., 2022). PACT, a dsRNA binding protein, activates RIG-I or MDA5, but the N proteins of PDCoV, MHV, and SARS-CoV directly bind to PACT, impeding the interaction between RIG-I/MDA5 and dsRNA or PACT, and thus inhibiting type I IFN activation (Chen et al., 2019). Moreover, the proteases PLpro and 3CLpro from MERS-CoV, SARS-CoV, and HCoV-NL63 inhibit multiple steps of type I IFN signal transduction by directly acting on signal transduction proteins or promoting their degradation through ubiquitin modification manipulations (such as interfering with IFN regulatory factor 3 and MAVS) (Barretto et al., 2005; Yang et al., 2014).

**Figure 1:**
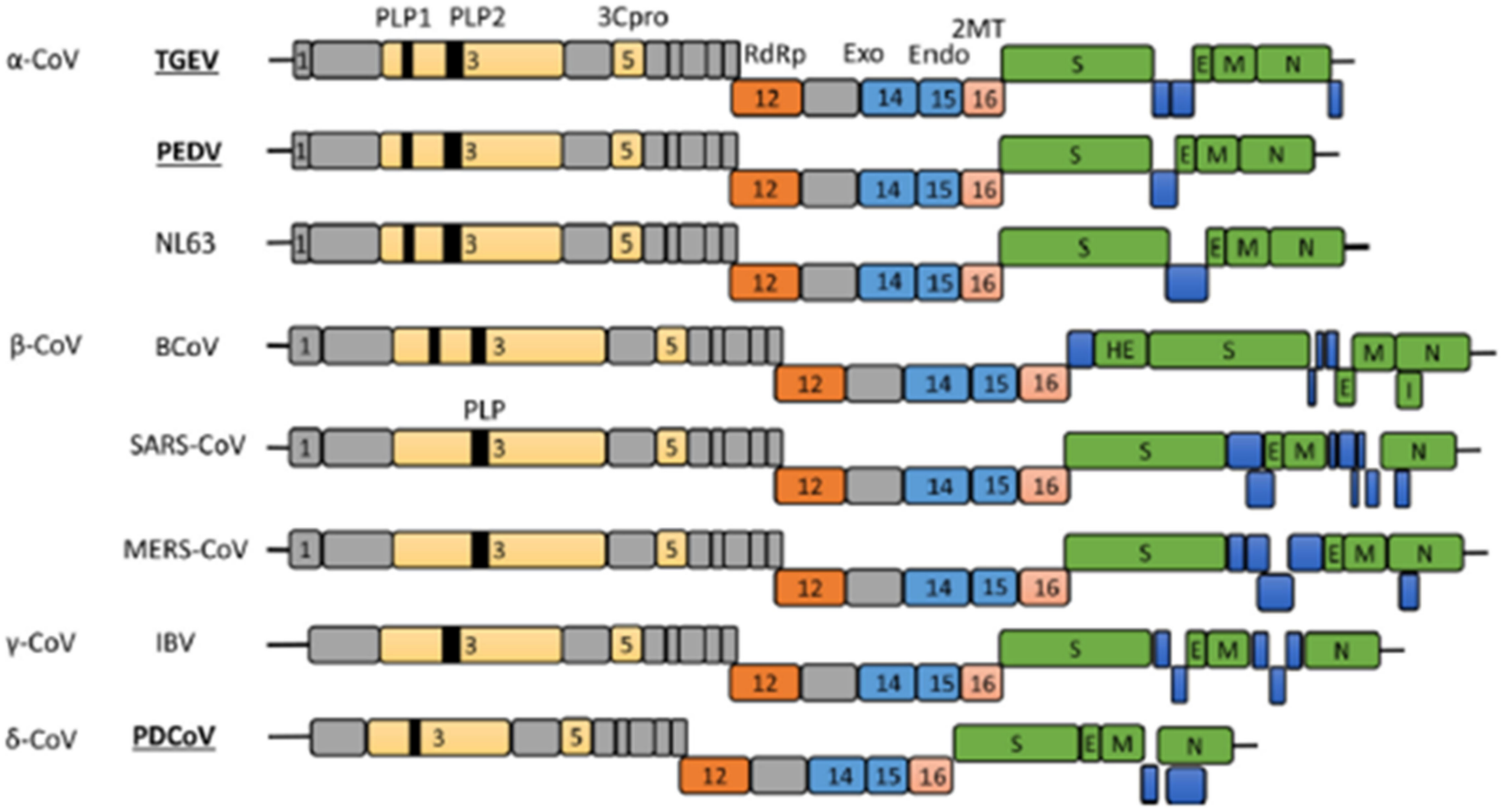
Coronavirus genome arrangement (Zhang and Yoo, 2016).

As the initial structural proteins in coronavirus replication, S proteins play a pivotal role in viral attachment and invasion. S proteins bind to specific receptors, including angiotensin-converting enzyme 2 (ACE2) (Li et al., 2003; Li et al., 2005; Zhou P. et al., 2020), aminopeptidase N (APN) (Tusell et al., 2007; Park et al., 2015), 9-O-acetylated sialic acid (Liu et al., 2015; Li et al., 2016), dipeptidyl peptidase 4(DPP4) (Li et al., 2017), and the carcinoembryonic antigen cell adhesion molecule (CEACAM1) (Dveksler et al., 1993), which facilitate the fusion between coronavirus membranes and host cell membranes, enabling viral entry into host cells. Furthermore, the S protein of coronaviruses is prone to amino acid mutations, leading to the emergence of novel strains with enhanced transmissibility and pathogenicity. There are 36 amino acid sites in the porcine epidemic diarrhea virus (PEDV) S protein that have a mutation frequency of more than 90%, and the high frequency of mutations makes it easier for the virus to escape from immune pressure, which may be the cause of recurrent outbreaks of piglet diarrhoea in pig farms (Yu et al., 2023). After the synthesis of viral subgenomic RNA, the membrane-associated structural proteins S, E, and M undergo translation and are inserted into the endoplasmic reticulum. From there, these proteins relocate to the endoplasmic reticulum-Golgi intermediate compartment. During this process, the N protein binds to the newly synthesized positive-sense RNA, forming a ribonucleoprotein complex. Following this, the M protein organizes the S, E, and N proteins into mature viral particles through protein-protein interactions, ultimately releasing them outside the cell via exocytosis (Fehr and Perlman, 2015; Hartenian et al., 2020). The E proteins, being the smallest structural proteins, primarily participate in virus assembly and budding (Masters, 2006; Nieto-Torres et al., 2011) (**Figure 1**).

## 2 Autophagy

Autophagy holds a pivotal role in cellular growth and development. As an evolutionarily conserved lysosome-reliant metabolic pathway within eukaryotic cells, autophagy functions to transport senescent or impaired organelles and proteins to the lysosome for degradation. The resulting degraded small-molecule products are then utilized in sustaining cellular material recycling and maintaining intracellular homeostasis (Rubinsztein et al., 2011; Wei et al., 2018; Levine and Kroemer, 2019). Furthermore, autophagy serves as a crucial component of the body’s defense mechanism, playing a vital role in resisting the invasion of pathogenic microorganisms. Upon viral invasion, cells rely on autophagy to transport viral proteins, nucleic acids, and other components to the lysosomes for degradation (Levine, 2005; Choi et al., 2018; Yang and Klionsky, 2020). Generally, autophagy encompasses three primary types: macroautophagy, microautophagy, and molecular chaperone-mediated autophagy.

Typical autophagy comprises several sequential stages: initiation, nucleation, structural extension of the phagocytic vesicle membrane to encapsulate the cargo, closure of the phagocytic vesicle membrane to form autophagosomes, fusion of autophagosomes with lysosomes to yield autophagolysosomes, and ultimately, the *in vivo* degradation of autophagic contents within the autophagolysosomes (Levy et al., 2017; Yin et al., 2019). The upstream signaling pathways of autophagy, Akt-mTOR and AMPK-mTOR, govern the afferent activation of autophagy signals. These signals are relayed to the autophagy initiation complex, the ULK (ATG1) complex, which comprises ULK1, ATG13, FIP200, and ATG101. Upon activation, the ULK complex proceeds to activate the VPS34 complex (composed of VPS34, VPS15, and Beclin-1). The VPS34 complex facilitates phagocytic vesicle membrane elongation via binding to ATG14, and subsequently, closure of the membrane to form autophagic lysosomes through the interaction with UVRAG. This binding mediates the maturation of autophagic vesicles and further closure of the phagocytic vesicle membrane to form autophagic lysosomes (Glick et al., 2010; Mizushima et al., 2011). ATG5-ATG12-ATG16L1 and LC3-II, as crucial membrane components of phagocytic vesicles, are recruited to the vesicle membrane during the membrane extension phase. Following phagocytic vesicle closure, the ATG5-ATG12-ATG16L1 complex detaches from the membrane (Klionsky et al., 2021). LC3-I serves as the precursor of LC3. Upon cleavage by ATG4, the C-terminal glycine residue is exposed and subsequently binds with phosphatidylethanolamine (PE) to form LC3-II. LC3-II is also recognized as a marker molecule for autophagosomes (Kirisako et al., 1999; Kirisako et al., 2000). Ultimately, the contents within the autophagic vesicles, after fusing with lysosomes, undergo degradation, and the resulting macromolecular precursors are recycled for reuse. Alongside LC3-II, the specific substrate of autophagy, P62, also plays a role in mediating selective autophagic processes, including mitochondrial autophagy. Therefore, the expression levels of LC3-II and P62 can serve as indicators, reflecting the extent of autophagic vesicle formation and the efficacy of autophagic lysosomes in degrading autophagic substrates (Klionsky et al., 2021).

Increasing evidence suggests that a diverse array of pathogenic microorganisms, encompassing viruses, have evolved numerous tactics to modulate host cell autophagy (Jackson, 2015; Lennemann and Coyne, 2015). The Influenza A virus (IAV) orchestrates autophagy by intricately modulating the AKT-mTOR signaling cascade and influencing the expression of HSP90AA1 via its NP and M2 proteins. This regulatory mechanism subsequently fosters the replication of IAV (Wang et al., 2019). Similarly, the capsid protein VP2 of the Foot-and-Mouth Disease Virus (FMDV) engages in a functional interaction with the heat shock protein HSPB1, thereby activating the EIF2S1-ATF4-AKT-MTOR pathway. This activation triggers a sequential cascade reaction that induces autophagy and ultimately enhances the replication of FMDV (Sun et al., 2018). Similarly, the Japanese encephalitis virus (JEV) facilitates the infection of host cells by suppressing autophagy. The decrease in autophagy-related proteins, namely ATG5 and ATG7, results in the accumulation of JEV RNA, an elevation in viral titers, and an augmentation in cell mortality. Notably, viral replication complexes co-localize with markers of ERAD regulation, specifically EDEM1 and the LC3-I. Furthermore, the downregulation of non-lipidated LC3 diminishes viral titers (Sharma et al., 2014). Although previous studies have predominantly focused on the interplay between human coronaviruses and autophagy, there is a scarcity of reports exploring the interactions between various classes of coronaviruses and autophagy. In this paper, we comprehensively review the interactions between different coronavirus species and autophagy, conducting a meticulous systematic study of coronavirus infections’ impact on autophagic processes and their underlying mechanisms.

## 3 α-CoVs

**Figure 2:**
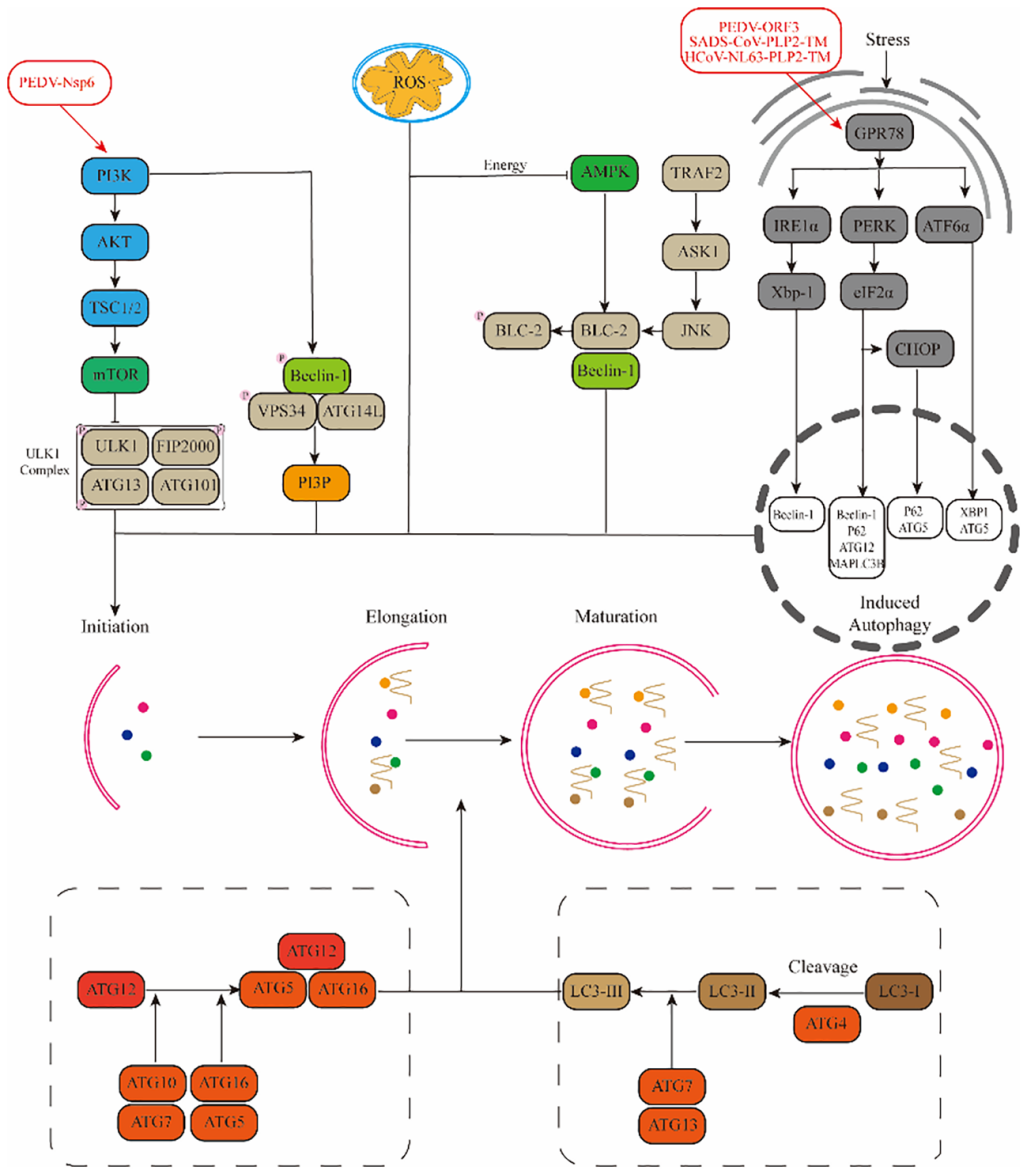
Mechanisms by which α-CoVs regulate autophagy through different signalling pathways.(1) Viruses promote autophagy through the PI3K-AKT-mTOR and AMPK-mTOR-ULK1 signalling pathways. (2) ROS regulate autophagy occurrence through downstream pathways. (3) Viruses promote autophagy through stress pathways: RE1α-XBP1 and PERK-eIF2α.

Among the research on alpha-coronaviruses (α-CoVs), there has been a more profound examination of PEDV, TGEV, SADS-CoV, and HCoV-NL63. Therefore, we have consolidated the mechanisms in which the pertinent proteins of these viruses regulate autophagy through diverse pathways (**Figure 2**).

### 3.1 PEDV

Porcine Epidemic Diarrhea Virus (PEDV), a coronavirus notorious for inducing vomiting, diarrhea, dehydration, and even mortality in pigs, exhibits a staggering prevalence of up to 100% among newborn piglets (Wood, 1977). Research has shown that PEDV infection in Vero cells can activate the autophagy pathway, leading to a downregulation in the phosphorylation levels of mTOR and its downstream effectors 4EBP1 and p70S6K (Guo X. et al., 2016). Further investigations revealed a substantial increase in the number of autophagic vesicles in PEDV-infected Vero cells, indicating a positive correlation between autophagy and NF-κB activation (Guo et al., 2017). A similar trend was observed in PEDV-infected IPEC-J2 cells, where autophagy activation promoted PEDV replication. However, Kong et al. presented a contrasting view, demonstrating that upon PEDV infection in Vero and LLC-PK1 cells, upregulated BST2 recruited the autophagy-associated cargo protein MARCH8 to catalyze the ubiquitination of the PEDV N protein. This process subsequently inhibited PEDV replication through the selective autophagic degradation of the N protein (Kong et al., 2020). Among the viral proteins of PEDV, Nsp3, Nsp6, and ORF3 are the primary inducers of autophagy activation. Nsp3 primarily inhibits the formation of autophagic streams by hindering the fusion of autophagic vesicles with lysosomes, while Nsp6 functions as an inhibitor of the PI3K/Akt/mTOR pathway to activate autophagy (Chen et al., 2014; Lin et al., 2020). On the other hand, the ORF3 protein upregulates GPR78 protein expression, activating the PERK-eIF2α pathway, triggering endoplasmic reticulum stress, and thereby activating autophagy (Zou et al., 2019).

### 3.2 TGEV

Transmissible Gastroenteritis Virus (TGEV) is an acute and highly contagious porcine enteric virus that infects pigs of various breeds and ages, resulting in symptoms such as vomiting, diarrhea, dehydration, intestinal villous atrophy, and high mortality rates among piglets (Turlewicz-Podbielska and Pomorska-Mól, 2021). Research has indicated that TGEV infection triggers an autophagic response in PK15 and ST cells, leading to an increase in monolayer and bilayer vesicle structures, which contribute to the formation of autophagic vesicles (Guo L. et al., 2016). Further investigations revealed that TGEV-induced autophagy may stem from the induction of mitochondrial autophagy by the N protein, which mitigates oxidative stress and apoptosis triggered by TGEV infection (Zhu et al., 2016).

### 3.3 SADS-CoV

Swine Acute Diarrhea Syndrome Coronavirus (SADS-CoV) is a novel porcine coronavirus that poses a significant threat to piglets, often resulting in severe mortality (Gong et al., 2017). In a study conducted by Zeng et al., the global gene expression profile of Vero cells infected with SADS-CoV was analyzed using RNA-seq, revealing a notable downregulation in the expression levels of PI3K and Akt genes (Zeng et al., 2021). Furthermore, it was discovered that SADS-CoV promotes autophagy in Vero cells by inhibiting the activation of the Akt/mTOR pathway (Zeng et al., 2022). Additionally, the Coronavirus-associated papain-like protease PLP2 (PLP2-TM) within SADS-CoV triggers endoplasmic reticulum stress through its interaction with GRP78 and subsequently induces autophagy via the activation of the IRE1-JNK-Beclin1 pathway (Shi et al., 2023).

### 3.4 HCoV-NL63

The Human Coronavirus (HCoV-NL63) also triggers autophagy. Specifically, PLP2-TM, through its interaction with Beclin1, enhances the accumulation of autophagosomes and impedes the fusion between autophagosomes and lysosomes, thereby inducing incomplete autophagy (Chen et al., 2014). Additionally, the non-structural protein Nsp3 of HCoV-NL63 relies on papain activity to induce autophagy but exerts an inhibitory effect on the autophagic flow (Chen et al., 2014).

In summary, both PEDV and TGEV infections are capable of stimulating the production of autophagic vesicles within susceptible cells, and the resulting autophagy represents a comprehensive autophagic process, commonly referred to as complete autophagy. A notable distinction arises in the specific interaction between BST2 and the N protein of these viruses; BST2 selectively engages with the N protein of PEDV to facilitate its autophagic degradation, whereas no such interaction occurs with the N protein of TGEV. Similarly, both the PLP2-TM domain of SADS-CoV and HCoV-NL63 have the ability to induce autophagy, but they differ in their outcomes. SADS-CoV triggers a complete autophagy process, where autophagosome accumulation is intimately linked to the hydrolysis of autophagic cargo. Conversely, HCoV-NL63 induces an incomplete autophagy, a process that ultimately impedes the successful maturation of autophagosomes into autophagolysosomes.

Recent investigations have unveiled intriguing differences in the replication mechanisms among various coronaviruses, further revealing that the influence of autophagy on these mechanisms varies significantly across cell types. Specifically, TGEV is capable of eliciting mitochondrial autophagy in IPEC cells, a process mediated by DJ-1, a versatile redox-sensitive protein. This autophagic response alleviates oxidative stress and apoptosis, thereby fostering viral replication (Zhu et al., 2016). In contrast, when TGEV infects PK-15 cells, the induced autophagy serves to impede viral replication. Analogously, PEDV induces autophagy in IPEC cells via the PI3K/AKT/mTOR signaling cascade, which in turn amplifies viral replication. However, when IPEC cells are treated with rapamycin, a potent autophagy inducer, it increases autophagic flux to a level that ultimately leads to the suppression of viral infection, illustrating the complexity and cell-specific nature of these interactions (Ko et al., 2017).

## 4 β-CoVs

**Figure 3:**
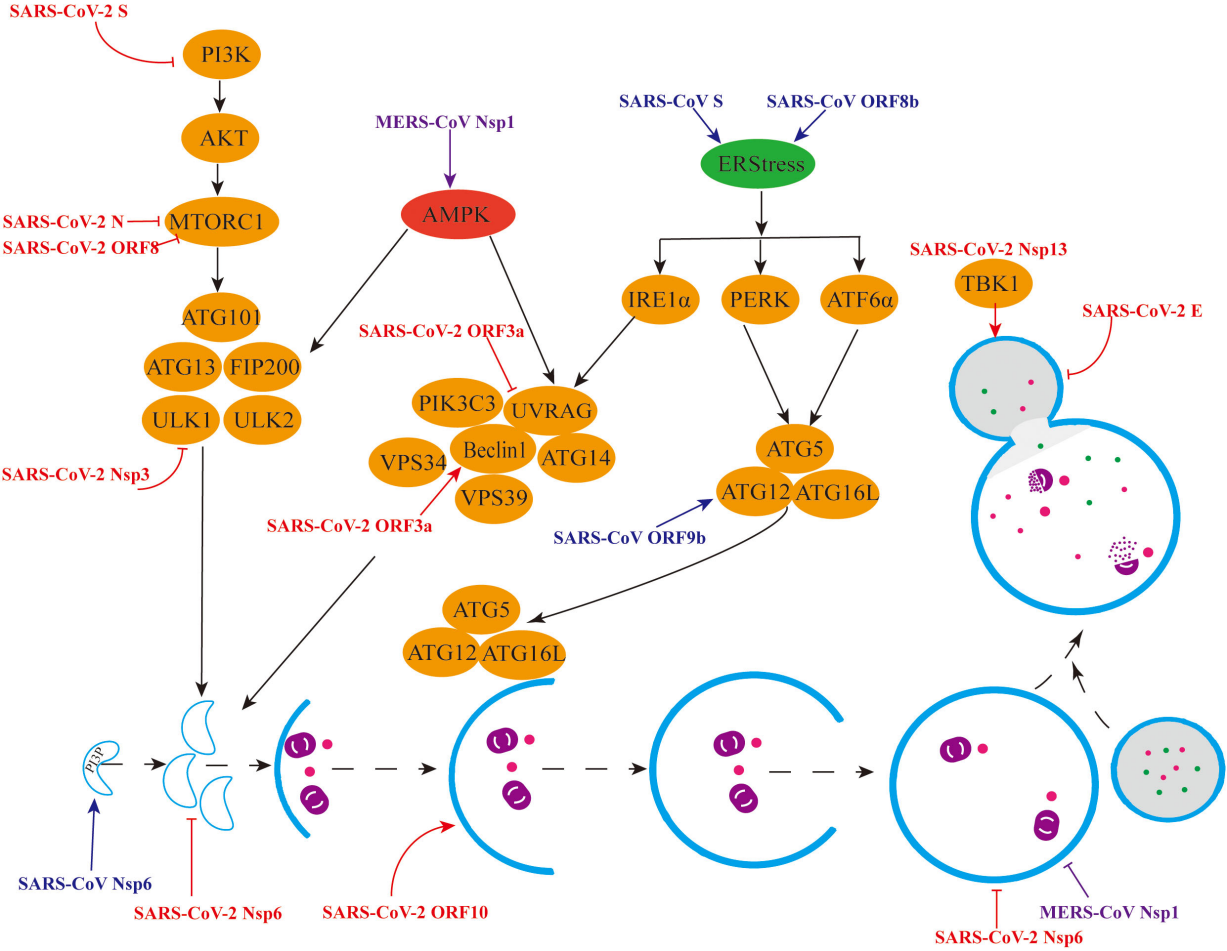
Mechanisms by which β-CoVs regulate autophagy through different signalling pathways. (1) Red colour indicates regulation of the autophagy pathway by individual SARS-CoV-2 viral proteins. (2) Blue colour indicates the promotion of autophagy by individual SARS-CoV viral proteins. (3) Purple colour indicates the different regulation of the autophagy pathway by the MERS-CoV Nsp1 protein.

In contrast to α-CoVs, the study of autophagy induced by β-CoVs is comparatively intricate, encompassing primarily SARS-CoV-2, SARS-CoV, MERS-CoV, MHV, and PHEV. We hereby provide a concise overview of the intricate interactions between β-CoVs and autophagy (**Figure 3**).

### 4.1 SARS-CoV-2

Since the global outbreak of Severe Acute Respiratory Syndrome Coronavirus 2 (SARS-CoV-2) in 2019, the epidemic has persisted, posing a significant threat to human and animal health. Due to its high priority, research on SARS-CoV-2, particularly in relation to autophagy, has been extensively conducted. Metabolomic analyses have revealed a significant upregulation of proteins associated with autophagy in SARS-CoV-2-infected cells, encompassing proteins involved in autophagy initiation (AMPK, TSC2, ULK1) and those critical for membrane nucleation and autophagy precursor formation (Beclin1, VPS34, ATG14) (Gassen et al., 2021). Furthermore, Krogan et al. demonstrated, through phosphoproteomic analysis using quantitative mass spectrometry, that SARS-CoV-2 infection promotes the activation of CK2 and p38/MAPK signaling pathways in Vero cells, leading to the production of various cytokines and the inhibition of the PI3K/Akt pathway, thus indicating that SARS-CoV-2 infection enhances autophagy (Bouhaddou et al., 2020).

Multiple proteins encoded by SARS-CoV-2 have been identified to modulate autophagy through diverse mechanisms. At the cellular level, the non-structural protein Nsp3 is capable of cleaving the autophagy initiator protein ULK1, thereby hindering the formation of the ULK1 complex and subsequently suppressing autophagy (Mohamud et al., 2021). Moreover, apart from inhibiting ULK1-mediated autophagy, the deubiquitination of Nsp3 may disrupt the cellular process of selective autophagy, ultimately fostering viral replication. Nsp6 functions to inhibit autophagy by interacting with VAMP7, ESYT2, ATP2A2, and TBK1, thereby suppressing the production of pre-autophagosomal structures (Kumar et al., 2021). Additionally, Nsp13 directly targets TBK1 and recruits it to P62 for autophagic degradation, thereby inhibiting type I IFN production (Sui et al., 2022). The ORF3a protein interacts with VPS39 and the HOPS complexes, inhibiting the fusion between autophagosomes and lysosomes, which subsequently impedes autophagic degradation. Notably, the accumulation of autophagy volume facilitates virus trafficking and excretion via lysosomal exocytosis (Chen et al., 2021). Conversely, during the autophagic degradation phase, ORF3a, ORF7a, and Nsp6 hinder autophagic flow by compromising lysosomal function, further suppressing autophagy (Miao et al., 2021).

The viral proteins of SARS-CoV-2 can also facilitate autophagy production. Specifically, the structural protein S binds to the receptor ACE2, stimulates ROS production, and inhibits the activation of the PI3K/Akt/mTOR pathway, thereby promoting autophagy (Li F. et al., 2021). Additionally, the structural protein E activates the ER pathway, leading to the phosphorylation of the translation initiation factor eIF-2α, which in turn promotes LC3 lipidation and induces autophagy (Waisner et al., 2023). The structural protein N interacts with LARP1 and inhibits the activation of the mTORC1 pathway, ultimately inducing autophagy (Gordon et al., 2020). ORF3a plays a dual role in autophagy, not only inhibiting autophagy production but also inducing it. This protein can interact with HMGB1 and induce RETREG1/FAM134B-mediated reticulophagy through the HMGB1-Beclin1 pathway (Zhang et al., 2022). Furthermore, the ORF8 protein induces the production of autophagic vesicles and degrades MHC-I via the Beclin1-mediated autophagy pathway (Zhang et al., 2021). Additionally, ORF8 interacts with the FKBP7 protein to inhibit the mTORC1 pathway, leading to the induction of autophagy (Gordon et al., 2020). Lastly, the overexpression of ORF10 promotes LC3 accumulation in mitochondria and induces mitochondrial autophagy by binding to the mitochondrial autophagy receptor NIX (Li et al., 2022).

Clinically, Ramachandran et al. observed that the viral proteins of SARS-CoV-2, including M, Nsp6, ORF3a, ORF9c, and ORF10, selectively target the endoplasmic reticulum and mitochondria, leading to autophagy in cardiomyocytes, suppression of mitochondrial function, disruption of intracellular Ca2+ homeostasis, decreased cell viability, and ultimately cellular death (Ramachandran et al., 2022). Li et al. uncovered through RNA sequencing analysis of peripheral blood mononuclear cells (PBMCs) from SARS-CoV-2-infected patients that the virus disrupts the expression of genes related to cellular stress responses (ER and HSF1) while also inducing autophagy-related genes. Notably, the virus-induced heat-shock response was intricately linked to autophagy (Li S. et al., 2021). Barbati et al. reported autophagy defects in PBMCs from SARS-CoV-2-infected patients, wherein the expression of LC3-II and p62 was significantly upregulated compared to normal cells. Moreover, the expression of these markers positively correlated with lymphocyte apoptosis and negatively correlated with lymphocyte counts, indicating that SARS-CoV-2-induced autophagy hijacking contributes to apoptosis (Barbati et al., 2022). Recently, LC3, SQSTM1, and BECN1 have emerged as potential new tools for differentiating patients with moderate to severe SARS-CoV-2 infection from asymptomatic individuals. Xia et al. found that patients with LC3 concentrations below 5.5 ng/mL should be hospitalized promptly for treatment (Fang et al., 2021). Conversely, BECN1 levels positively correlated with disease severity in SARS-CoV-2 patients, exhibiting significantly higher levels compared to the normal group, thus indicating its potential as a marker for assessing SARS-CoV-2 disease severity (Okuyan et al., 2021).

### 4.2 SARS-CoV

In 2003, China reported a devastating respiratory disease, known as Severe Acute Respiratory Syndrome Coronavirus (SARS-CoV), which posed a significant threat to human health. By April 4th, 2003, a total of 2,353 cases had been documented, resulting in a mortality rate of approximately 4 per cent (Lee et al., 2003). SARS-CoV has been found to induce autophagy, and the viral proteins it encodes can modulate this process through diverse mechanisms. Wileman et al. discovered that the non-structural protein Nsp6 is capable of inducing LC3 to produce autophagosomes. Moreover, Nsp6 recruits the effector protein DFCP1 by upregulating the expression of PI3P, which subsequently facilitates the nucleation of detached membranes (Cottam et al., 2011). Remarkably, the diameter of Nsp6-induced autophagosomes is notably smaller compared to those induced by starvation (Cottam et al., 2014). Furthermore, research has demonstrated that SARS-CoV infection is associated with selective autophagy in mitochondria, and the ORF9b protein specifically induces autophagy through the classical autophagic pathway (Shi et al., 2014).

SARS-CoV infection disrupts the functionality of the endoplasmic reticulum, subsequently triggering an unfolded protein response (UPR). Notably, the S proteins encoded by SARS-CoV stimulate the activation of diverse UPR transcription factors, such as GRP78, GRP94, and C/EBP homologues (Chan et al., 2006). Additionally, the ORF3a protein plays a pivotal role in lysosomal membrane permeabilization, promoting the release of lysosomal tissue proteases, which leads to lysosomal impairment and dysfunction (Yue et al., 2018). Moreover, the lysine residue at position 77, which is dependent on the ORF8b protein, forms insoluble intracellular aggregates. These aggregated ORF8b proteins induce endoplasmic reticulum stress and lysosomal damage, ultimately enhancing autophagic flow through the nuclear translocation of TFEB (Shi et al., 2019).

### 4.3 MERS-CoV

In 2012, Saudi Arabia reported a highly pathogenic coronavirus known as the Middle East Respiratory Syndrome coronavirus (MERS-CoV), which is transmitted from dromedary camels to humans (De Wit et al., 2016). Kindrachuk et al. conducted a peptide kinome array and functional network analysis on human hepatocytes infected with MERS-CoV, revealing that MAPK/ERK1/2 and PI3K/Akt/mTOR signaling pathways were specifically regulated. This suggests that MERS-CoV infection may have implications on autophagy processes (Kindrachuk et al., 2015). Furthermore, Gassen et al. observed an accumulation of autophagosomes in Vero cells infected with MERS-CoV. Notably, the SKP2 protein promotes BECN1 ubiquitination, leading to proteasomal degradation of BECN1, thereby inhibiting the fusion of autophagosomes with lysosomes and ultimately impeding autophagy flow (Gassen et al., 2019). Additionally, the non-structural protein Nsp1 enhances ROS expression in cells, activating the MAPK pathway and inhibiting the mTOR pathway, thereby inducing autophagy (Feng et al., 2022). However, Nsp1 also inhibits lysosomal acidification, which further impedes autophagic flow (Feng et al., 2022).

### 4.4 MHV

Mouse hepatitis virus (MHV) pioneered as the first viral model to delve into the intricate correlation between coronaviruses and autophagy, specifically for investigating viral replication mechanisms and immune responses (de Haan et al., 2005). While Prentice et al. observed that MHV triggers autophagy and necessitates ATG5 to bolster viral replication (Prentice et al., 2004). Zhao et al.’s findings offer a contrasting perspective, revealing that MHV can replicate in BMM cells devoid of ATG5, thereby indicating that the autophagy process induced by MHV is not reliant on ATG5 (Zhao et al., 2007).

### 4.5 PHEV

Porcine hemagglutinating encephalomyelitis virus (PHEV), a virus that has the potential to impact the nervous and digestive systems of pigs, triggers symptoms such as vomiting, anorexia, profound lethargy, ataxia, and ultimately leads to the demise of infected pigs within 2-3 days of disease onset (Roe and Alexander, 1958). Li et al., utilizing neuroblastoma cells with ULK1 knockout, discovered that the formation of PHEV-induced autophagosomes does not necessitate the participation of ULK1, indicating that PHEV-induced autophagy represents a non-canonical process, independent of the AMPK-mTORC1-ULK1 signaling pathway (Li Z. et al., 2021). Ding et al. further revealed that PHEV infection in Neuro-2a cells induces autophagy, with the production being temporally dependent. However, PHEV infection impedes the fusion between autophagosomes and lysosomes, thereby inhibiting the progression of autophagic flux (Ding et al., 2017).

Since the outbreak of SARS in 2003, emerging coronaviruses have garnered extensive attention, with various coronavirus proteins exhibiting distinct mechanisms in activating or inhibiting autophagy processes, as illustrated in **Table 2**. Nevertheless, numerous coronaviruses remain understudied, resulting in a profound lack of comprehension regarding the intricacies of autophagy in the context of coronavirus infections and related pathologies. Consequently, the intricate relationship between autophagy and coronaviruses necessitates further exhaustive exploration.

**Table 2:** Betacoronavirus proteins regulate autophagy.

Betacoronavirus	Proteins	Function	References
SARS-CoV	ORF3a	Induces lysosomal damage and dysfunction	(Yue et al., 2018)
	ORF8b	Induces lysosomal damage and dysfunction	(Shi et al., 2019)
	Nsp6	Induce autophagosome production	(Cottam et al., 2011)
SRAS-CoV-2	ORF3a	Block autolysosome formation; Impair lysosomal function	(Miao et al., 2021)
	ORF8b	Initiate autophagy	(Gordon et al., 2020)
	Nsp6	Impairment of lysosomal function and inhibition of autophagy	(Kumar et al., 2021)
MERS-CoV	Nsp1	Induce autophagy or block autophagic flux	(Feng et al., 2022)
	Nsp6	Inhibits autophagy	(Gassen et al., 2019)

## 5 γ-CoV

### 5.1 IBV

Infectious bronchitis virus (IBV), a coronavirus that targets avian species, poses a significant threat to the poultry industry, causing a reduction in the productivity of laying hens and broilers, resulting in considerable economic losses (Broadfoot et al., 1956). There is compelling evidence that IBV exhibits intricate interactions with autophagy. Cottam et al. uncovered that the non-structural protein Nsp6 triggers autophagosome formation and facilitates the fusion of autophagosomes with lysosomes via the activation of class III PI3K (Cottam et al., 2011). Concurrently, Majer et al. discovered that IBV stimulates the formation of autophagosomes, which, in turn, amplifies autophagic signaling in Vero cells (Maier et al., 2013).

## 6 δ-CoV

### 6.1 PDCoV

In 2012, a novel porcine deltacoronavirus, designated PDCoV, was initially documented in Hong Kong (He et al., 2020). Upon infection with PDCoV, the virus induced the formation of double membrane vesicles and rearrangements of vesicle membranes, with a notable increase in the number of autophagosome-like vesicles detected within the cytoplasm. These observations hint that PDCoV might induce autophagy (Qin et al., 2019). In a proteomics-based study, Zhou et al. examined PDCoV-infected porcine small intestinal epithelial cells (IPEC-J2) and discovered that autophagy-related signaling pathways, including PI3K-Akt and mTOR, were activated during the infection process. This suggests that PDCoV triggers autophagy in IPEC-J2 cells (Zhou X. et al., 2020). Additionally, PDCoV infection in LLC-PK1 cells activates the p38 signaling pathway, ultimately leading to complete autophagy, which facilitates viral replication (Duan et al., 2021).

## 7 Concluding remarks

To date, a total of eight human coronaviruses and 13 animal coronaviruses have been reported. Coronaviruses are capable of inducing cell death in infected cells via diverse pathways, including apoptosis, necrosis, and autophagy (Yue et al., 2018). Despite the existing literature on the interplay between viruses and autophagy, the intricate mechanism of how viruses harness autophagy to facilitate their replication remains to be comprehensively understood. Additionally, autophagy can significantly influence cellular biological processes, and autophagic cell death, as a mode of programmed cell death, not only directly contributes to cell demise but also interacts with necrosis, apoptosis, ferroptosis, and other modes of cell death to expedite cell mortality (Gao et al., 2016). Whether these biological processes induced by autophagy have an impact on the replication cycle of coronaviruses merits further investigation.

In this review, our primary focus is on the viral regulation of autophagy and the intricate mechanisms underlying autophagy-virus interactions. However, several challenges persist that hinder our ability to gain a deeper understanding of the nexus between autophagy and coronavirus infection, necessitating further exploration. Firstly, the current research landscape on coronaviruses and autophagy mechanisms lacks the requisite experimental conditions. Specifically, a significant portion of the experimental data, particularly those pertaining to highly pathogenic coronaviruses, have been derived primarily from cellular models, leaving room for debate on whether these findings align with the outcomes of *in vivo* infection studies. Secondly, the impact of autophagy on viral replication remains contentious, a complexity that may be attributed to variations in cell type, strain type, and the timing of infection. Lastly, whether coronaviruses can trigger other forms of autophagy, such as chaperone-mediated autophagy (CMA) and microautophagy, remains an open question that demands further scrutiny.

In the context of the interplay between SARS-CoV-2 and autophagy, a range of drugs that modulate autophagy have been developed to target SARS-CoV-2 infection (Min et al., 2020; Hui et al., 2021; Yuen et al., 2021). Notably, Chloroquine, which functions as an inhibitor of the autophagy pathway, has been proposed as a biological treatment option for SARS-CoV-2 infection. For animals, autophagy plays a regulatory role in growth, reproduction, and production performance, to a certain extent, contingent upon the timing and degree of autophagy induction (Tesseraud et al., 2021). Recent studies have revealed that specific autophagy-related components and vesicles play a crucial role in coronavirus infection and replication. Furthermore, considering the diverse expression levels of autophagy-related genes and the abundance of autophagic vesicles across different cell types, alternative and complementary pathways may potentially enable the resumption of infection and replication processes after autophagy inhibition (Sun et al., 2023). Notably, high levels of autophagy inhibition or activation can lead to various cellular dysfunctions, including excessive endoplasmic reticulum stress and acute inflammatory responses. These findings suggest that conventional autophagy inhibitors may not effectively hinder SARS-CoV-2 infection or alleviate acute inflammatory responses. Instead, combination therapy involving autophagy-related drugs and other medications may yield superior therapeutic outcomes. Specifically, targeting specific autophagy mechanisms or organelle components in combination with other drugs may represent a promising new direction for the development of autophagy-based treatments for COVID-19 in the future.

In summary, autophagy emerges as a potential target in the treatment of coronavirus infections, and a comprehensive understanding of its interactions with coronaviruses is crucial for future therapeutic advancements.
